# Extracellular *vesicles* biogenesis, isolation, manipulation and genetic engineering for potential *in vitro* and *in vivo* therapeutics: An overview

**DOI:** 10.3389/fbioe.2022.1019821

**Published:** 2022-11-04

**Authors:** Nastaran Hadizadeh, Diba Bagheri, Mehdi Shamsara, Michael R. Hamblin, Abbas Farmany, Mengdi Xu, Zhuobin Liang, Farideh Razi, Ehsan Hashemi

**Affiliations:** ^1^ Diabetes Research Center, Endocrinology and Metabolism Clinical Sciences Institute, Tehran University of Medical Sciences, Tehran, Iran; ^2^ Department of Molecular Genetics, Tarbiat Modares University, Tehran, Iran; ^3^ Department of Animal Biotechnology, National Institute of Genetic Engineering and Biotechnology, Tehran, Iran; ^4^ Wellman Center for Photomedicine, Massachusetts General Hospital, Harvard Medical School, Boston, MA, United States; ^5^ Dental Research Centre and Dental Implant Research Centre, Hamadan University of Medical Sciences, Hamadan, Iran; ^6^ Shenzhen Bay Laboratory, Institute of Molecular Physiology, Shenzhen, China; ^7^ School of Biology and Biological Engineering, South China University of Technology, Guangzhou, China; ^8^ Metabolic Disorders Research Center, Endocrinology and Metabolism Molecular—Cellular Sciences Institute, Tehran University of Medical Sciences, Tehran, Iran

**Keywords:** Exosome, therapeutics, manipulation, extracellular vesicles, biogenesis

## Abstract

The main goals of medicine consist of early detection and effective treatment of different diseases. In this regard, the rise of exosomes as carriers of natural biomarkers has recently attracted a lot of attention and managed to shed more light on the future of early disease diagnosis methods. Here, exosome biogenesis, its role as a biomarker in metabolic disorders, and recent advances in state-of-art technologies for exosome detection and isolation will be reviewed along with future research directions and challenges regarding the manipulation and genetic engineering of exosomes for potential *in vitro* and *in vivo* disease diagnosis approaches.

## 1 Introduction

**FIGURE 1:**
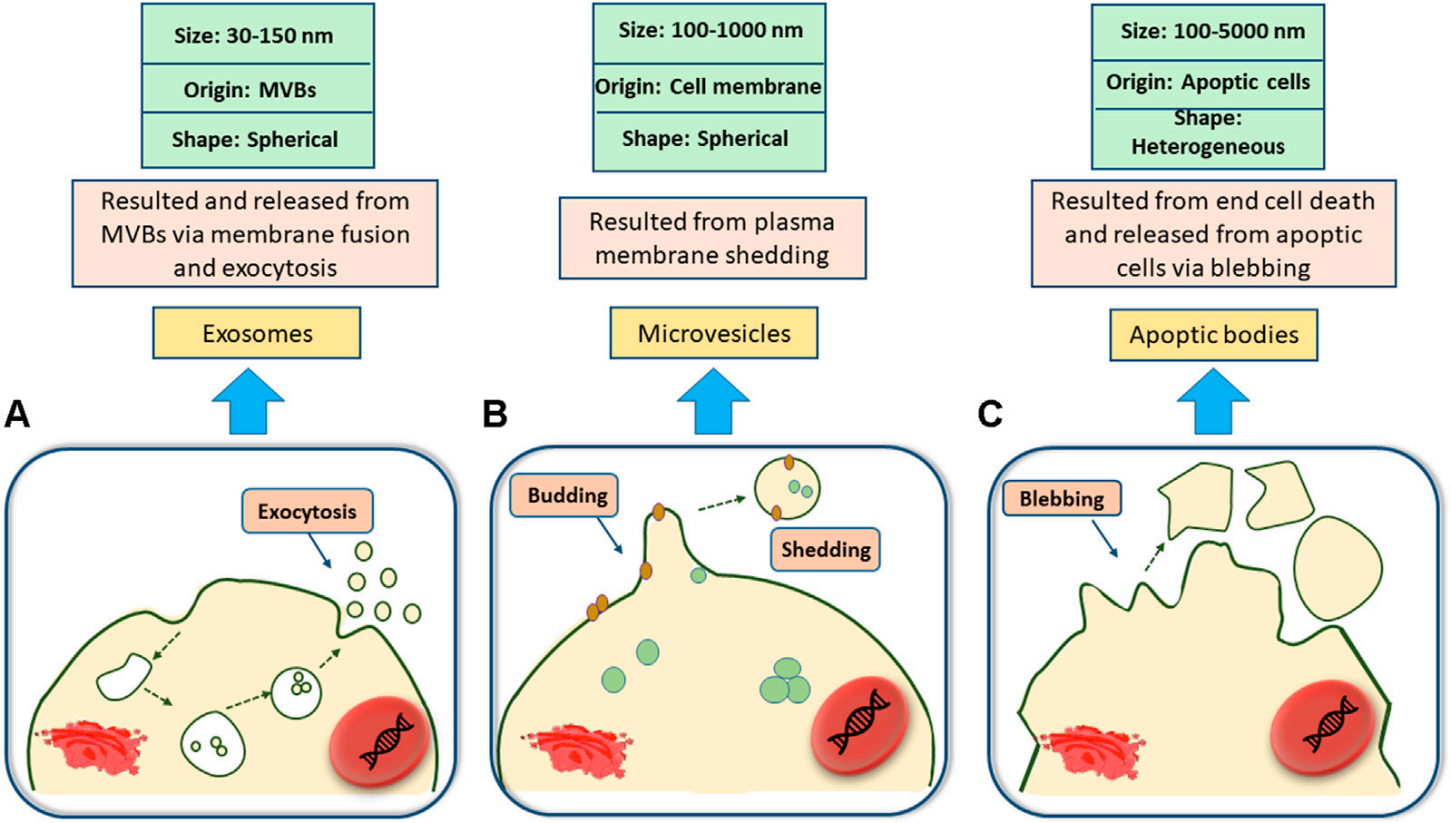
Classification of Extracellular vesicles. **(A)**; Exosomes are spherical nanosized biovesicles that are released via exocytosis as a result of multivesicular bodies (MVBs) fusion with the cellular membrane. **(B)**; Microvesicles are 100–1,000 nm spherical vesicles secreted from the outward budding and shedding of the plasma membrane. **(C)**; Apoptic bodies, ranging from 100 to 5,000 nm, may come in a diverse range of sizes and are produced upon the outward blebbing of the plasma membrane of dying and apoptic cells.

Being released from the surface of a wide range of eukaryotic cell types (such as mast cells, epithelial and endothelial cells, dendritic cells, astrocytes, and so on), extracellular vesicles (EVs) are nanoscale vesicles that are often found in extracellular spaces (Laulagnier et al., 2004; Valadi et al., 2007; Conde-Vancells et al., 2008; Zhang et al., 2015a; Fei et al., 2015; Bosque et al., 2016; Yaoyu et al., 2018) and can be isolated from cell culture mediums (Balaj et al., 2011) and different body fluids including blood plasma (Ashcroft et al., 2012), saliva (Michael et al., 2010), amniotic fluid, milk and even urine (Masyuk et al., 2010; Keller et al., 2011; Rupp et al., 2011; Théry, 2011). EVs are heterogeneous in types and are classified based on their size, originated cells, morphological characteristics, and distinctive functions (Gholizadeh et al., 2017). Small EVs or exosomes (50–120 nm), micro-vesicles (MVs) (0.1–0.35 µm), and apoptotic bodies (0.8–5 µm) (Théry et al., 2002; Moldovan et al., 2013; Raposo and Stoorvogel, 2013; Yáñez-Mó et al., 2015) are the main subsets of EVs and each has their own specific properties and functions. For instance, apoptotic bodies are produced by cells that are involved in programmed cell death (Yáñez-Mó et al., 2015), MVs are formed from the outer budding and shedding of the plasma membrane (Raposo and Stoorvogel, 2013), and exosomes are nano-sized vesicles originating from the interior side of the budding of late endosomal structures (also called multivesicular bodies (MVBs) (see Figure 1), that are released upon exocytosis pathways as a result of MVB fusion with the cellular membrane (Théry et al., 2002; Moldovan et al., 2013).

Small EVs, also known as exosomes, are one of the most fascinating subtypes of EVs that were previously considered to be extracellular debris for a long time before novel scientific advancements and the development of new methodologies expanded our understanding of exosomes as nanosized bioactive cargo-containing vesicles that play mediatory roles in intercellular communication, biological signal transduction, cellular behavior regulation, and immunomodulatory responses. Structurally, EVs are composed of a fluid core enclosed by a phospholipid bilayer (Jayabalan et al., 2017) and contain a remarkable amount of sphingomyelins and gangliosides. Fluid cores of EVs are similar to the cytosol of their primitive mother cells and are enriched with a variety of proteins (such as cytoskeletal proteins, and Tetraspanins), some of which may determine the distinct functionalities of exosomes new (Cocucci and Meldolesi, 2015; Meldolesi, 2016; Stremersch et al., 2016). Exosomes’ inherent carrier-like properties have enabled them to deliver a diverse range of molecules to their target cells and hence take part in different cellular pathways such as inflammation, carcinogenesis, cellular homeostasis, survival, transport, and regeneration (Jayabalan et al., 2017). Owing to their exquisite roles and cell-specific contents, exosomes have recently attracted tremendous attention in the fields of gene and drug delivery, non-invasive prognosis and diagnosis, vaccine development, and precision medicine.

Despite the outstanding advantages of exosomes as advanced diagnostic and therapeutic platforms, their evident mediation in promoting impaired cellular signaling pathways and inflammatory responses through the transduction of metabolic disorder signals and molecules to distant sites provides them with intrinsic dual-faced properties that may potentially lead to the progress of various pathological conditions such as insulin resistance, obesity, metabolic syndrome, diabetes type I and II, as well as life-threatening diabetic vascular complications (Guay et al., 2019; Dini et al., 2020; Chen et al., 2021).

Current literature regarding small EVs’ precise roles and potentials as well as their double-edged nature suffers from a lack of sufficiency, and a more thorough understanding of exosomes’ physiology, biogenesis, functions, and physiochemical characteristics is crucial for its exploring this topic on a deeper level. For this aim, this study was conducted to provide a comprehensive overview on exosome biogenesis, isolation, and manipulation with a shifted focus on the impact of exosomes in both the progress and treatment of metabolic disorders.

## 2 Small EVs characterization and properties

It has been more than 3 decades since small EVs or exosomes were first discovered and reported as vesicles with 5′ nucleotidase function (Théry et al., 1999). These phospholipid bilayered vesicles are one of the most important subtypes of EVs that possess a density of 1.13–1.19 g/ml (Chahar et al., 2015) and are secreted from a diverse range of eukaryotic cells including mast cells (MC) (Johnstone et al., 1991), dendritic cells (DC) (Van Niel et al., 2001), reticulocytes (Raposo et al., 1996), epithelial cells (Fauré et al., 2006), B-cells (Caby et al., 2005), and neural cells (Street et al., 2012).

Exosomes can be isolated from a variety of biofluids including plasma (Admyre et al., 2007), urine (Admyre et al., 2003), breast milk (Keller et al., 2007), bronchoalveolar lavage fluid (Brand et al., 2014), amniotic fluid (Madison et al., 2014), saliva (Street et al., 2012), semen (Lotvall and Valadi, 2007), and cerebrospinal fluid (Katsuda et al., 2013) in both physiologically normal and pathological conditions.

**FIGURE 2:**
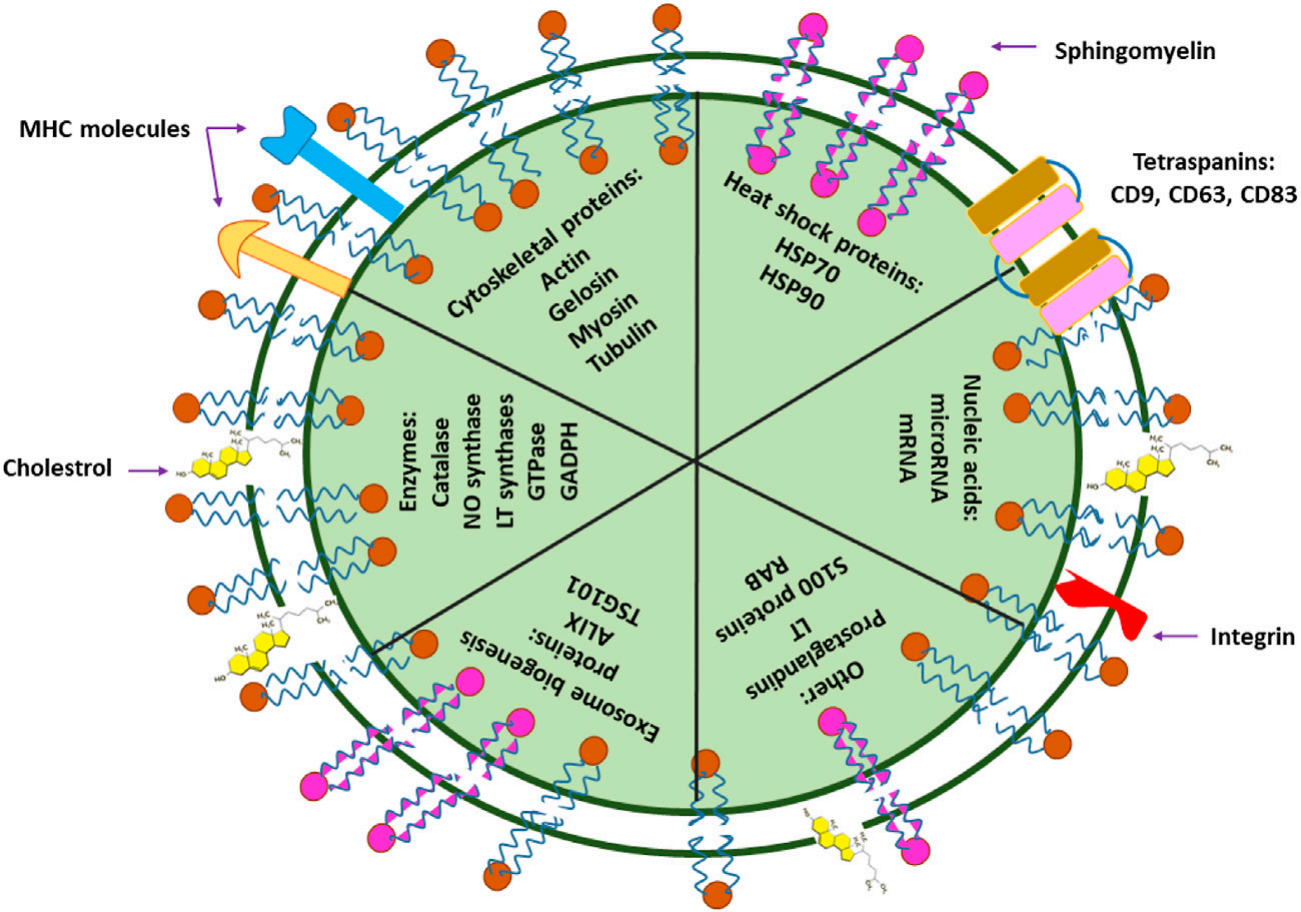
Small EV components. Exosomes contain nucleic acids (DNA and RNA), lipids (cholesterol, sphingomyelin), MHC (major histocompatibility complex; a group of immunologically-important, protein-encoding genes that exist on the surface of cells or small EVs, and assist the immune system in recognizing foreign particles), and different types of proteins. Proteins of small EVs are classified into numerous groups, some of which include cytoskeletal proteins (such as actin, gelosin, myosin, and tubulin), heat shock proteins (such as HSP70 and HSP90), biogenesis-assisting proteins (such as ALIX and TSG101), membrane proteins (such as tetraspanins) and enzymes (such as catalase, GADPH, GTPase, NO synthase). The components of small EVs such as their protein or nucleic acid types largely depend on their derivated parent cells and may differ in component types and abundance.

Similar to the plasma membrane, transmission electron microscopy (TEM) images of exosomes have illustrated exosomes as spherical structures enclosed by a lipid bilayer membrane (Zhang et al., 2016; Gholizadeh et al., 2017). However, in contrast to the plasma membrane, exosome membranes are enriched with cholesterol and sphingomyelin and possess poor amounts of lecithin and phosphatidyl ethanolamine (Subra et al., 2010). Accordingly, such differences in membrane components grant exosomes the advantage of enhanced stability as a result of increased membrane fluidity (Zhang et al., 2015a). Moreover, the remarkable quantity of lipid rafts (such as cholesterol and GM1 gangliosides) in exosome membranes is a critical factor that plays a major role in their formation (Figure 2) (Valapala and Vishwanatha, 2011).

Proteins are yet another crucial component of exosome membranes that may differ in type and abundance based on the constituents of their primary mother cells. For instance, integrins, tetraspanins (CD63, CD81, CD9, and CD82) (Record et al., 2011; Fujita et al., 2016), flotillin (Mears et al., 2004), annexin (Valapala and Vishwanatha, 2011), and heat shock proteins (hsp70 and hsp90) (Mathew et al., 1995; Campanella et al., 2012) are some of the important proteins that may often be exploited as distinguishing markers for the characterization and identification of exosomes with different origins (Figure 2).

Recent studies have shown that exosomes can transfer various proteins, lipids, DNA, RNA, and microRNA content (Campanella et al., 2012; Fei et al., 2015). In fact, the molecular content of exosomes is sometimes referred to as the fingerprint of their primitive cells and their physiological conditions (Van Giau and An, 2016). As a result, exosomes can be isolated and utilized as precise biomarkers that reflect the pathophysiological conditions of their originated cells to a notable extent (Skog et al., 2008; Kahlert et al., 2014; Melo et al., 2015). Due to their less invasive collection methods from natural body fluids and the detailed information they provide regarding the state of their originated cells, analysis of exosomal components is believed to be a valuable tool for facilitated prognosis and diagnosis of various diseases such as cancer (Hoshino et al., 2015; Melo et al., 2015; Wang et al., 2015), inflammation (Peinado et al., 2016), metabolic disorders (Dini et al., 2020), and cardiovascular diseases (Lawson et al., 2016). In addition, improved speed and cost-effectiveness of sample collection (blood, saliva, or urine collection) are some of the other contributing factors that have turned exosomes into preferable and beneficial platforms for disease diagnosis (Vlassov et al., 2012), prevention, prediction, and providing potentially effective treatment for certain diseases (Lee et al., 2016).

### 2.1 Morphology and size

There are many different methods for the characterization of exosomes such as scanning electron microscopy (SEM), transmission electron microscopy (TEM), and cryo-EM (Zhang et al., 2018). Due to the small diameters of exosomes (30–150 nm), electron microscopy (EM) methods are essential for their characterization as they cannot be detected by optical characterization approaches (Van Der Pol et al., 2010). While these methods are commonly utilized for exosome characterization, they both require prior sample ultracentrifugation as well as sample pre-processing (Wu et al., 2015). Interestingly, a study revealed that the size of exosomes characterized by SEM and TEM were rather similar, however, their morphological shapes were different (cup-shaped and spherical for TEM and SEM-characterized exosomes, in a respective order). With the general consensus considering exosomes as spherical structures, it is believed that the additional sample pre-processing steps for TEM including gradient dehydration, heavy metal-assisted contrast straining, and cellulose embedment are responsible for the morphological variations observed in TEM-characterized exosomes (György et al., 2011; Raposo and Stoorvogel, 2013).

### 2.2 Contents

Serving as a common criterion for their classification, small EVs’ contents help to predict their clinical diagnosis values and therapeutic potentials. These heterogeneous vesicles are rich in lipids, proteins, and different types of nucleic acids including DNA or RNA (mRNA, microRNA, and other types of noncoding RNA) (Balaj et al., 2011; Ashcroft et al., 2012). The originated cells from which small EVs are released are often regarded as the main root of their distinct properties and roles, as those primary mother cells determine the small EV’s contents and bioactive cargo, including their membrane lipid elements (such as sphingomyelin, cholesterol, ceramide, and so on) and protein subtypes (Michael et al., 2010; Keller et al., 2011). Besides their function-distinguishing roles, the significance of small EVs’ components also resides in the existing biomarkers that small EVs carry as an inheritance from their parent cells. When isolated and analyzed, these biomarkers have the ability to reveal the parent cell’s inner condition and display any abnormal pathophysiological conditions on a cell-scale level (Salomon et al., 2015; Willms et al., 2016; Zhang et al., 2019). The properties of small EVs can be discovered by using different methods such as traditional western blotting, enzyme linked immunosorbent assay, mass spectrometry, polymerase chain reaction, and sequencing techniques (Masyuk et al., 2010; Rupp et al., 2011). Furthermore, specific compositions of EVs’ surfaces can be detected by labeling EVs with antibody-coated gold nanoparticles and performing immune electron microscopy under TEM (Théry, 2011).

### 2.3 Density

Density is a key contributing factor to EV isolation. The density of microvesicles is still unknown, while the density of exosomes has been measured to about 1.13–1.19 g/ml (Michael et al., 2010). Even though this remains beyond the scope of this review, it is noteworthy to mention that various approaches help to determine EVs’ density, for instance, Olcum *et al.* developed a nanomechanical resonator that measures EVs’ weight, thus with the additional knowledge of EVs’ size, their density can be easily calculated (Zhang et al., 2019).

## 3 Biogenesis and release signals

**FIGURE 3:**
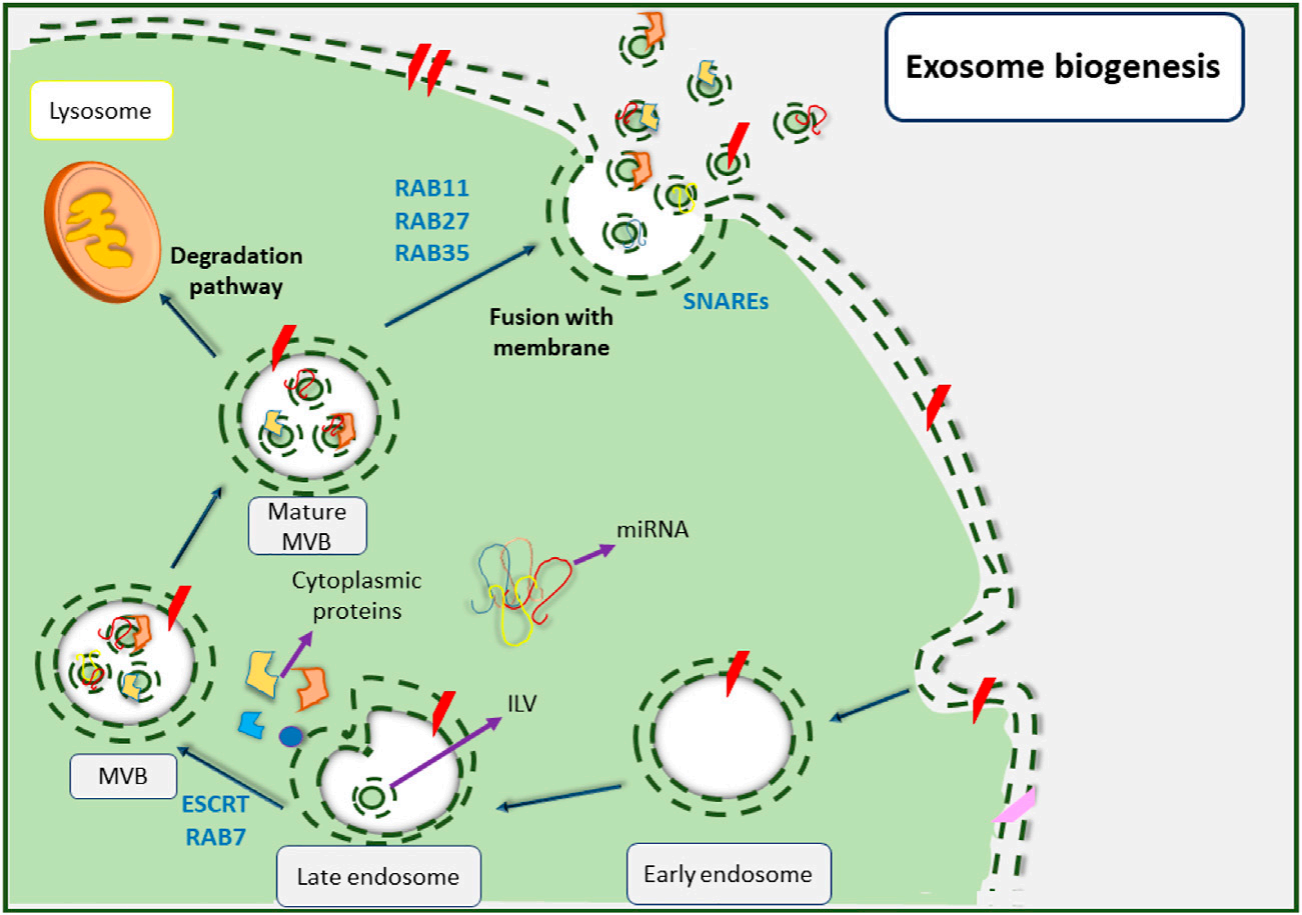
Small EVs biogenesis pathway. Biogenesis of small EVs is initiated upon the formation of early endosomes as a result of cellular membrane inward invagination. Followed by that, late endosomes are shaped before their membrane begins to invaginate inward into the luminal space to create nanoscale intraluminal vesicles (ILVs). The late endosomes also change to multivesicular bodies (MVBs) that now contain ILVs. Next, MVBs are matured and can follow two different fates; being degraded upon combination with lysosomes, or fusing with the cellular membrane and releasing small EVs into extracellular spaces.

The generation and release of small EVs are essentially complicated processes that depend on various factors. Biogenesis of exosomes begins with the invagination of the plasma membrane from areas that are affluent of phosphatidylserine (Figure 3) (Lee et al., 2016). At first, an early endosome shapes from the inside of a budding via endocytosis of the plasma membrane under the control of intracellular calcium concentration (Dignat-George and Boulanger, 2011; van der Pol et al., 2012; Colombo et al., 2014). In this stage, annexin plays a key regulatory role (Tan et al., 2013). This early endosome undergoes several alterations before changing to a late endosome (Zhang et al., 2016). Subsequently, the late endosomal membrane begins to invaginate inward into the luminal space and creates nano-sized vesicles (30–100 nm in diameter) called intraluminal vesicles (ILVs) that reside inside multivesicular bodies (MVBs) or previously called late endosomes (Stoorvogel et al., 1991; Keller et al., 2006; Stuffers et al., 2009; Akers et al., 2013). Various proteins are involved in these events, for instance, flotillin participates in the invagination of the membrane, and soluble *N*-ethyl maleimide sensitive factor attachment protein receptors (SNAREs) complexes play an important role in the fusion of MVBs with the surface of target cells (Mears et al., 2004). The MVB biogenesis pathway also requires various proteins including Tsg101, Alix, tetraspanins (CD-63, CD-9, CD-82, and CD-81), RAB, GTPases, Annexins (membrane fusion proteins), cell adhesion molecules, growth factor receptors, and heat shock proteins (HSP-70 and HSP-90) (Mathew et al., 1995; Clayton et al., 2004; Campanella et al., 2012).

Depending on the endosomal sorting complex that is required for transportation (ESCRT), MVBs can either choose to degrade by combining with lysosomes or can form exosomes by becoming fused to the plasma membrane. The function of the ESCRT complex is necessary for the development of exosomes (Kowal et al., 2014; Liu et al., 2017) as it is involved in membrane remodeling and also facilitates the formation of intraluminal vesicles (ILVs) (Colombo et al., 2013).

The ESCRT complex consists of four sub-complexes including ESCRT-0 which takes part in the recruiting of ubiquitinated proteins for cargo protein selection, ESCRT-I and ESCRT-II which trigger the membrane to form buds hence help the formation of ILVs, and ESCRT-III which induces vesicle secretion. Some other proteins such as VPS4, VTA1, and ALIX also exist in the ESCRT complex (Colombo et al., 2014; Liu et al., 2017; Frankel and Audhya, 2018). It is also important to mention that some studies have reported an ESCRT-independent pathway that is capable of forming MVBs without relying on the ESCRT complex subunits (Müller, 2012; Kranendonk et al., 2014). Eventually, MVBs are merged with the plasma membrane upon maturation and are then released into the extracellular space by RAB and GTPase protein activities (Müller, 2012; Kranendonk et al., 2014).

It is well-established that the secretion mechanism of small EVs does not take place by chance and involves many detailed cellular and molecular pathways, most of which are currently unexplored as this field still remains in its infancy. Increased exosome secretion can be influenced by various factors such as changes in pH, hypoxia, oxidative stress and thermal shocks, and radiation (Stuffers et al., 2009). Exosome release can also take place under the influence of certain molecules such as p53 which is increased in stressful conditions (Savina et al., 2002).

Based on the above-mentioned information, it can be concluded that exosomes have different characterizations and features depending on their cell origins. Hereon, we will discuss some of the biophysical and biochemical features of small EVs as well as different isolation methods based on their characterizations.

## 4 Conventional and advanced small EV isolation methods

**FIGURE 4:**
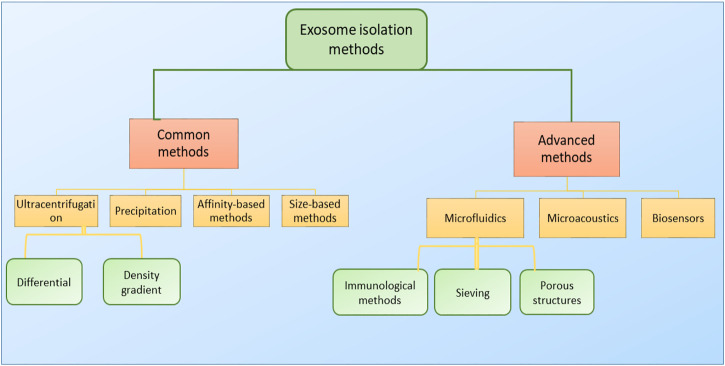
Classification of small EV isolation methods. Small EVs are often isolated via two main classes of isolation methods (common and advanced). Common or traditional methods include ultracentrifugation, precipitation, affinity-based methods, and size-based methods, and mainly rely on small EVs’ physiochemical properties for their isolation. Advanced methods, as ever-improving technologies, include microfluidic approaches, microacoustic methods, and biosensors. Each one of these platforms may possibly consist of several methods for exosome isolation, and some platforms may take advantage of combining two or more techniques into one isolation platform to promote isolation and increase the system’s efficiency.

For the isolation of small EVs, different factors including time, cost, and purity of the extracted EVs should be taken into consideration. The physical and biochemical properties of EVs are major factors in EV isolation, as most isolation methods are based on density, surface contents, size, or precipitation (Figure 4). Density-based methods consist of ultracentrifugation and density-based gradient centrifugation. There are also several high-tech approaches that benefit from higher exosome purity while being more cost-effective at a large scale. To this day ultracentrifugation, as a density-based method, is considered to be the gold standard for small EV isolation (Bosque et al., 2016) and accounts for 56% of all exosome isolation techniques used in research (Cocucci and Meldolesi, 2015). Due to requiring little technical expertise, little or no sample pre-preparation, and fewer expenses, ultracentrifugation is considered as a user-friendly method (Meldolesi, 2016). However, due to the necessitation of special equipment and consuming prolonged periods of time, as well as providing low exosome purity and inefficient exosome yield (5%–25% recovery) (Stremersch et al., 2016), it is not of satisfactory sufficiency to be exploited in clinical environments, especially in cases of emergency.

### 4.1 Common isolation methods

#### 4.1.1 Differential ultracentrifugation

**FIGURE 5:**
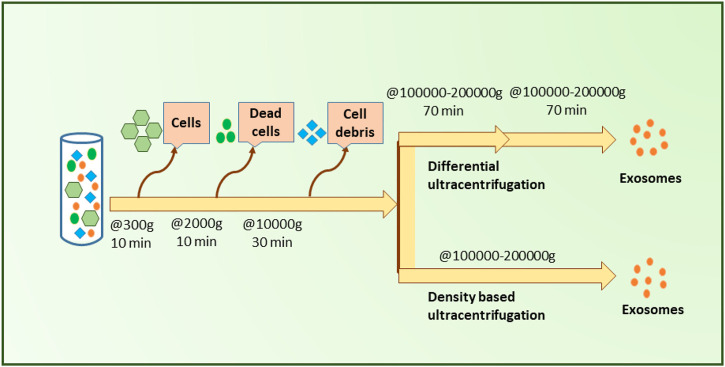
Ultracentrifugation-based small EVs separation. As illustrated, differential centrifugation and density-based centrifugation both acquire a few sample pre-processing steps in order to remove cells, dead cells, and cell debris from the initial blood sample and facilitate the separation of small EVs more accurately. Upon the removal of blood cells and cell debris, samples need to be centrifuged at high forces (✕100000–200000 g) twice for differential centrifugation. In contrast, density-based ultracentrifugation requires only one round of centrifugation at a similar high force (✕100000–200000 g) to that of differential ultracentrifugation.

Differential ultracentrifugation is based on the sequential removal of particles based on their size and density through different centrifugal durations and forces (Livshts et al., 2015). This method provides proper exosome purity, which is why it is known as the gold standard technique for exosome isolation (Figure 5) (Li et al., 2017). The process in this method is carried out gradually and step by step and follows the order of separating cells, cell debris, apoptic cells, and microvesicles. Throughout this process, as the remaining particles become smaller, the rotation speed is increased sequentially (Sidhom et al., 2020). Differential ultracentrifugation accounts for more than 80% of exosome isolations before 2015 (Gardiner et al., 2016; Théry et al., 2018), however, a shift of popularity has been taken note of as potential pitfalls of this method were revealed with time. Forming exosomal aggregates (Linares et al., 2015), long duration, labor intensiveness, and the need for highly costly equipment can be noted as major disadvantages of this method (Street et al., 2012; Szatanek et al., 2015; Yu et al., 2018; Ebert and Rai, 2019).

#### 4.1.2 Density gradient ultracentrifugation

Density gradient ultracentrifugation isolates exosomes based on their size, mass, and density. A density gradient medium with gradually increasing density from top to bottom is utilized to increase the final exosome purity in this technique (Li et al., 2017; Sidhom et al., 2020).

While this method remains one of the most widely used methods to this day, it provides low exosome yield, requires a lot of time, and requires expertise as well as expensive equipment (Lobb et al., 2015; Konoshenko et al., 2018) (see Figure 5).

#### 4.1.3 Size-based methods

Size-based methods such as membrane filtration and size exclusion chromatography are popular size-based methods used for exosome separation (Sidhom et al., 2020).

##### 4.1.3.1 Ultrafiltration

The basis of filtration relies heavily on pore-consisting membranes that permit exosomes to pass through the gaps, whereas bigger sample components such as cells, cell debris, and macromolecules are not allowed to pass through the pores (Zhang et al., 2020). In this approach, larger particles are removed by the utilization of filters with 0.8 and 0.45 µm pore dimensions. Followed by the collection of the remaining sample, smaller particles are then eliminated by a filter with pores of smaller diameters compared to exosomes (0.22–0.1 µm). It is clear that in this method, exosomes are obtained between the filters with maximal and minimal pore sizes (Théry et al., 2018). This method provides the advantage of being low in costs as well as not altering the activity of exosomes. However, low purity rate, low non-specific binding of exosomes to membranes, and low exosome recovery are among the reasons that filtration is not suitable for wide usage (Vergauwen et al., 2017; Zhang et al., 2020).

##### 4.1.3.2 Size exclusion chromatography

Size exclusion chromatography (SEC), also known as gel filtration, is a size-based method in which an aqueous solution is used as the mobile phase that carries the sample along the column, and a porous filtration polymer is used as the stationary phase that allows differential elution. In this method, different sample components are separated based on their size, as larger particles are eluted prior to smaller ones, however, other factors such as molecular weight and shape can also affect this separation process. SEC has been previously adapted in a single-step process for exosome isolation and takes about 20 min to carry out (Böing et al., 2014). This feature, along with its cost-effectiveness and preservation of exosome integrity, morphology and functionality are accounted for circumventing some of the major limitations of ultracentrifugation (Sidhom et al., 2020). However, it is important to note that low exosome purity, yield, and a lack of specificity in isolating exosomes from particles of almost the same size, are known as the major drawbacks that prevent this method from being utilized as the optimal technique for exosome isolation (Gámez-Valero et al., 2016).

#### 4.1.4 Exosome precipitation

Exosome precipitation is another EVs isolation method that can be used to facilitate the isolation process, shorten the required duration, and also ensure small EV isolation with a relatively high yield. By altering the solubility or dispersibility of exosomes, exosomes can be forced out of biological fluids. For this matter, water-excluding polymers should be used without a need for any special equipment (Street et al., 2012). Therefore, this technique is suitable for clinical usage and is scalable for large sample sizes (Meldolesi, 2016). A recently published article has reported a precipitation technique for isolating exosomes. This method uses an aptamer/AuNP biosensor for colorimetric profiling of exosomal proteins. This biosensor consists of a gold nanoparticle (AuNP) with a panel of aptamers. The complex of aptamers prevents the nanoparticles from aggregating at high salt solutions. Normally, in the absence of exosome, the aptamer and AuNP are bound together with a weak bond, but in the presence of exosomes, this bond breaks, and a stronger bond forms between the aptamer and exosome surface proteins and subsequently leads to the aggregation of AuNP (Lotvall and Valadi, 2007).

#### 4.1.5 Affinity-based exosome isolation

Another common method for exosome isolation is the affinity-based isolation method. In this approach, antibodies are usually used against the surface markers of exosomes (mostly tetraspanins). Immunoaffinity-based isolation of exosomes can also be done via incubation of the samples with magnetic beads (Koliha et al., 2016), or with FeO_3_ nanocubes loaded and coated with antibodies against exosomal surface proteins (Boriachek et al., 2019). This process can also be carried out for antibodies against exosome-adhesion molecules such as heat shock proteins (Ghosh et al., 2014), heparin (Balaj et al., 2015), and epithelial cell adhesion molecules (Brunnermeier and Palia, 2020). This method provides high-purity exosomes and is an excellent technique to selectively isolate exosomes that carry certain biomarkers; however, it results in lower exosome yield as not all exosomes in a sample might carry a specific marker. To mention one of the drawbacks of this technique, the potential loss of exosome integrity can be stated. This, however, only occurs in conditions where antibodies cannot be easily removed from the exosomes (Reiner et al., 2017). Different characteristics of the utilized antibodies, such as their specificity, quality, and price can draw some limitations regarding the widespread usage of this technique.

### 4.2 Advanced isolation methods

Aside from the aforementioned methods, other new and advanced methods for exosome isolation have been reported which are usually based on microfluidic chips and immunological separation technologies.

#### 4.2.1 Microfluidic-based isolation techniques

Microfluidic techniques are new isolation methods that are in their early stages of development. They are proven to be fast, rapid, and efficient in terms of product purity.

These methods can be classified into three categories:

i) Immunoaffinity-based methods
ii) Sieving methods
iii) Using porous structures for trapping exosomes (Wang et al., 2013).

##### 4.2.1.1 Immunological separation methods

The first immunoaffinity-based method was proposed by Chen et al. (2010) and is capable of capturing exosomes on a microfluidic chip. The main principle of this approach mostly relies on the receptors from the outer membrane of exosomes that enable specific collection from lipid structures and other membrane-shed particles based on their functions and origin cells. Chen et al. (2010) proposed another method concerning the previous technologies. This method works faster, consumes about 1 h of time, and requires fewer reagents (100–400 µl). With the help of this method and the utilizatoin of CD63 (common exosome marker), a total of 30 ng RNA was captured on-chip) from 400 µl samples.

Using the same principle, Kanwar et al. (2014) designed an on-chip exosome quantification device called “Exochip”, which uses a fluorescent-based method on a standard read-out plate reader. In order to increase the mixing rate, this device is also featured with several circular wells that are connected to each other by a narrow channel. Exochip requires more time which results in a stronger interaction between exosomes and the surface. By adding more wells to this device, it is also possible to scale up its function. The protein and nucleic acid yield from this device is 15–18 μg and 10–15 ng in 400 µl of a serum sample, respectively. A higher level of fluorescence on the chip was measured in exosomes that were obtained from patients with pancreatic cancer, in comparison with healthy controls (Kanwar et al., 2014).

Of note, it is important to take into consideration that immunological techniques are advanced methods that are used in pure exosome isolation.

##### 4.2.1.2 Sieving

Recently, a new and different approach was proposed by Davies et al., 2012 that relies on sieving exosomes from whole blood. In this method, exosomes are mechanically sieved through a membrane using either electricity or pressure. Due to the less negative charge of proteins, if electrical fields are used for sieving, more purified exosomes will be obtained. Pressure-driven methods help us to achieve results much faster, however, a drawback of this technique is the exosome damage that can be caused.

##### 4.2.1.3 Trapping exosomes in a porous microstructure

Wang et al. (2013) designed a porous microstructure for traping exosomes. This design consists of a ciliated micropillar structure that forms a microporous silicon nano-wire with the ability to selectively trap particles with a size range of between 40 and 100 nm diameter. They explained that this step is rather fast (10 min) (Wang et al., 2013). However, the whole process is quite time-consuming as the nano-wire has to be dissolved in PBS buffer overnight. The highest retention (60%) was obtained for 83 nm vesicles when up to 30 µl of the sample was used, followed by 120 nm vesicles (45%), while the retention of 500 nm bead was only 10%. This device was not used for clinical samples and no analysis was performed on RNA and cargo protein.

All of the explained methods have their own benefits and drawbacks. Regarding time, immunological techniques take a relatively short time (1.5 h) to separate exosomes with high specificity. Trapping exosomes in porous microstructures is rather time-consuming (1 day), but has the advantage of providing high exosome recovery. On the other hand, the sieving technique has the benefit of working with whole blood; however, it provides low exosome recovery and can damage vesicles due to the high rate of stress that is applied. Moreover, there are other isolation techniques for microvesicles that might also be suitable for exosome isolation (Chen et al., 2010; Kanwar et al., 2014). It is estimated that a combination of these methods and other techniques such as ultracentrifugation, can be used in the future to improve total throughput as well as exosome recovery efficiency. An advantage of sieving in comparison with other methods is its ability to work on whole blood, while the other methods need off-chip sample preparation steps such as reagent mixing or plasma extraction (Davies et al., 2012; Kanwar et al., 2014).

#### 4.2.3 Microacoustics

Acoustic-based approaches are some of the newest techniques for exosome isolation and can lead to high exosome yield and make-up for some of the issues of previous isolation methods. Earlier, acoustic-based isolation techniques required additional steps for sample preprocessing and thus needed extra equipment and time. Also, they could only separate two types of targets and were unable to separate exosomes from complex and multicomponent fluids such as undiluted blood. Recently Wu et al., 2017 designed a new on-chip device for isolating exosomes from biological fluids such as blood, urine, and saliva by using acoustics waves. This technique is a single-step combination of microfluidics and acoustic waves that leads to exosome isolation with relatively fast speed, high exosome yield, recovery, and almost no human involvement. The process is mostly automated, label-free, contact-free, compatible with nature, and provides us with information about the isolated exosomes’ structures, functions, and characteristics.

## 5 Small EV loading

Small EVs can be directly loaded with therapeutic agents by employing several types of non-viral delivery methods. These methods are mainly divided into two categories, including passive and active encapsulation methods.

### 5.1 Passive cargo-loading methods

#### 5.1.1 Incubation of drugs with exosomes or donor cells

Loading drugs into small EVs by simple incubation is a common route exploited in some recent drug delivery studies. In this technique, the drug molecule is added to the cell culture medium at therapeutic concentrations and is then incubated with exosome-secreting cells. In another approach, exosomes are first isolated from their secreting cells and are then incubated with certain therapeutic molecules. The molecular size of the exploited drugs must be small enough to pass through the exosome membrane (Johnsen et al., 2014), however, the efficacy of this incubation method is rather low. To enhance the drug loading capacity, exosomes might be treated with membrane permeabilizers such as saponin that bind to the cholesterol in cell membranes to form pores (Fuhrmann et al., 2015). Saponin has previously been applied for loading catalase and hydrophilic molecules into exosomes (Podolak et al., 2010).

### 5.2 Active cargo-loading methods

#### 5.2.1 Electroporation

Electroporation is one of the most frequently used methods to transfect mammalian cells. Different types of therapeutic materials including DNAs, RNAs, and proteins have been encapsulated into small EVs by electroporation in the past. A mixture of the drug of interest and exosomes is initially prepared in a conductive solution before being delivered to exosomes by applying an electrical field. The molecules are then diffused into small EVs through the small pores created in their lipid membrane upon the application of an electrical pulse (Johnsen et al., 2014). This method is widely used for loading exosomes with molecules such as siRNAs and miRNAs that are not capable of being diffused into exosomes through the lipid membrane due to their large dimensions (Wahlgren et al., 2012). Despite the notable advantages offered by this technique, the administration of electroporation for loading drugs into small EVs suffers from several limitations, for instance, low loading efficiency which is resulted from nucleic acid precipitation and exosome structural instability (Kooijmans et al., 2013; Lamichhane et al., 2015). To solve this problem, Johnsen *et al.* developed an optimized electroporation buffer using trehalose disaccharides to improve the small EVs’ stability and decrease their aggregation (Johnsen et al., 2016).

#### 5.2.2 Sonication

In this method, drug molecules are loaded into exosomes by sonication. A mechanical shearing force is first generated by a sonicator probe which destabilizes the exosome membrane and allows the diffusion of drugs into exosomes. It is also crucial to note that in this technique, the membrane instability is not damaged permanently, and the lipid bilayer integrity and surface-bound proteins are restored by the incubation of exosomes at 37°C (Kim et al., 2016).

#### 5.2.3 Direct drug conjugation to exosomes

Drug molecules can be covalently bound to the surface of exosomes. In this regard, copper-catalyzed azide-alkyne cycloaddition, known as click chemistry, has been observed to efficiently conjugate small molecules to exosome surfaces. In principle, click chemistry reacts between an alkyne group and an azide one to form a triazole linkage. Smyth *et al.* successfully conjugated the alkyne cross-linked exosomes to azide-fluor (Smyth et al., 2014). The mild condition of this bioconjugation does not affect the exosome size and its internalization into the target cells.

#### 5.2.4 Using antibodies against exosomal proteins

Exosomes contain proteins in their bilayer lipid membrane that are derived from their parental cells. They are characterized by the expression of specific protein markers such as CD9, CD63, and CD81 on their surface (Andreu and Yáñez-Mó, 2014). These protein markers are important in the recognition of target cells as well as the transportation of exosomes within the cells. Moreover, different types of molecules such as fluorescent dyes that are conjugated to antibodies can be bound to these surface proteins and be used for tracking exosomes *in vivo*. Yamada et al., 2013 used anti-CD9 antibodies coupled with magnetic beads for exosome isolation from cattle milk. Tumor-derived exosomes, which are actively secreted by tumor cells, are a potential source of cancer biomarkers. Immunoaffinity-based isolation of melanoma cell-derived exosomes was performed on the plasma of patients using a specific antibody for the CSPG4 epitope, which is specifically expressed in melanoma cells (Sharma et al., 2018).

## 6 Genetic engineering of exosomes

Genetic engineering of exosomes is performed by genetic manipulation of donor cells followed by isolation of the exosomes that contain desired genetic materials or drugs. Donor cells are transfected by viral and non-viral vectors with the most efficient method that delivers genetic materials into cells (Koppers-Lalic et al., 2013).

### 6.1 Exosome surface modification

The surface of exosomes can be engineered by modifying the proteins that are emerged on the exosome membrane. Exosome surface engineering can be applied as a targeting strategy for the delivery of therapeutic drugs to desired cells. For instance, exosomes derived from cardiosphere-derived cells (CDCs) can stimulate angiogenesis, induce cardiomyocyte proliferation and modulate cardiomyocyte apoptosis and hypertrophy. To generate an efficient exosomal delivery system to target cardiomyocytes, the CDC was manipulated to express Lamp2b, an exosomal membrane protein, which is fused to a cardiomyocyte-specific peptide (CMP). Targeted exosomes resulted in increased uptake by cardiomyocytes, decreased cardiomyocyte apoptosis, and higher cardiac retention following intramyocardial injection when compared to non-targeted exosomes (Mentkowski and Lang, 2019).

### 6.2 Exosome content engineering

Exosomes are inherently loaded with their parent cell-derived molecules such as different lipids, peptides, proteins, and nucleic acids. Researchers are now constantly trying to take advantage of this opportunity to load exosomes with desired therapeutic molecules via genetic engineering of the primary-secreting cells. MicroRNAs (miRNAs) are a class of small, non-coding RNAs with a length of 19–22 nts that suppress gene expression post-transcriptionally as they bind to their target mRNA’s 3′–untranslated regions via base complementarity. Once bound, the mRNA of interest is either silenced or degraded, henceforth is not translated to a protein. MiR-126 is an important regulator of endothelial cell (EC) function and angiogenesis. To investigate the effect of miR-126 on amelioration of ischemia/reperfusion (I/R)-injured EC function, Pan et al., 2019 transfected mesenchymal stem cells (MSCs) with a miR-126 short hairpin RNA and isolated miR-126-loaded exosomes. Treatment of I/R-injured ECs with the engineered exosomes resulted in enhancement of survival and angiogenic function in injured ECs and subsequently activated the PI3K/Akt/eNOS pathway, decreased the expression of cleaved caspase-3, and also increased angiogenic and growth factors.

## 7 Small EVs as emerging therapeutic delivery platforms

Besides their intrinsic stability and remarkable loading capability, small EVs benefit from low immunogenicity and the ability to penetrate through biological barriers. Such advantages have in turn granted exosomes with exceptional delivery values, making them suitable candidates for drug and gene delivery purposes (Song et al., 2021).

A previous study has indicated that exosome-loaded curcumin is associated with greater anti-inflammatory effects compared to free curcumin in mice as a result of enhanced plasma curcumin concentrations (Sun et al., 2010). Paclitaxel, as a common therapeutic agent for cancer, has been incorporated inside macrophage-derived exosomes and exhibited significantly greater toxic effects in drug-resistant cancer cells (Kim et al., 2017). Aminoethylanisamide-polyethylene glycol has also been transferred inside small EVs loaded with paclitaxel and used to specifically target sigma receptors in lung carcinogenic cells. These platforms displayed improved drug uptake and accumulation in cancer cells (Kim et al., 2018). Serving as an efficient antioxidant enzyme, catalase has been encapsulated inside small EVs and administered intranasally to the brain cells of mice with Parkinson’s disease, where it showed notable uptake by neurons (Haney et al., 2015).

Small EVs have the ability to prevent miRNA degradation by shielding them from nucleases in blood circulation. Interestingly, evidence suggests that small EV-assisted delivery of therapeutic nucleic acids not only contains higher efficiency but also benefits from reduced cytotoxicity in contrast to traditional transfection approaches (Momen-Heravi et al., 2014; Johnsen et al., 2016; Kobayashi et al., 2020). the miRNA-155 inhibitor has been successfully delivered by small EVs from B cells in order to reduce the production of TNF-α in macrophages (Momen-Heravi et al., 2014; Zhao et al., 2020). A tumor suppressor called miR-199a-3p was loaded inside ovarian cancer fibroblasts-derived small EVs and displayed promising results for the treatment of ovarian cancer (Kobayashi et al., 2020). CRISPR/Cas9 is a novel technology utilized to carry out genome editing investigations due to its excellent flexibility and precision (Li et al., 2020a). Despite such advantages, the use of carrier agents that deliver CRISPR/Cas9 is often limited due to low immunogenicity. Owing to their enhanced immunogenicity and carrier properties, small EVs have recently attracted notable attention as CRISPR/Cas9-carrying vehicles. For instance, tumor-derived exosomes filled with poly (ADP-ribose) polymerase-1 (PAPR-1) have been successfully applied to inhibit the proliferation of cancer cells (Kim et al., 2017). The same technique was applied for targeting hepatic stellate cells in hepatic fibrosis through exosome-assisted delivery of CRISPR-dCas9-VP64 (Luo et al., 2021). In a recent study, Lin and coworkers designed a hybrid liposome-exosome system to load and deliver CRISPR/Cas9 components and observed that the encapsulated nucleic acid was endocytosed and expressed by mesenchymal stem cells (Lin et al., 2018). Despite their valuable potential for the delivery of therapeutic agents, small EVs suffer from a lack of targeting ability. Several studies have been constructed to ameliorate this characteristic, for instance, miRNA-140-containing small EVs were first incorporated with chondrocyte-affinity peptides and administered intra-acularly to deep cartilage tissues, and eventually prevented the progressionof osteoarthritis (Liang et al., 2020). Nucleic acid nanoparticles (NANPs) are promising therapeutic platforms comprised of multiple nucleic acids. Due to the existing challenges in the delivery of NANPs, exosomes, as natural nanoscale carriers, have recently gained significant attention for improved NANPs delivery (Ke and Afonin, 2021). In an interesting study, complex nucleic acid nanoparticles (NANPs) containing several therapeutic nucleic acids (such as RNA cubes and RNA rings) were designed and delivered to GFP (Green Fluorescent Protein) cells through exosome-mediated delivery. NANP-loaded exosomes were successfully internalized inside targetted cells where they activated NF-kB decoy and RNAi (RNA interference) pathways and eventually silenced targeted genes (Nordmeier et al., 2020). This study also concluded that RNA and DNA fibers were recognized interdependently, activated NF-kB decoys, and diminished the production of pro-inflammatory cytokines.

## 8 Exosomes’ immunomodulation properties and immunotherapeutic applications

Being released from almost all cell types and playing a dynamic role in intercellular communication, exosomes have recently been extensively investigated as novel immunotherapeutic agents with the ability to regulate various immune responses (Kugeratski and Kalluri, 2021; Dai et al., 2022; Dutta and Paul, 2022). Research has revealed that exosomes exhibit immunoregulatory or immunostimulatory effects through different pathways (Olejarz et al., 2020; Benjamin-Davalos et al., 2021; Kugeratski and Kalluri, 2021). Owing to their immunomodulatory properties, exosomes have recently attracted significant attention regarding their potential as immunotherapeutic agents that can be used in organ transplantation and anti-cancer therapies (Zheng et al., 2021). End-stage organ failure is one of the foremost factors leading to organ transplantation. While this procedure is considered to save the lives of many all around the world, it is associated with increased long-term mortality as well as reduced quality of life in patients (Russell et al., 2020; Vanholder et al., 2021). Long-term survival of transplanted organs is a major issue in transplant procedures which requires life-long consumption of immunosuppressant medicine such as glucocorticoids to prevent possible rejection reactions in the host’s body (Dobbels et al., 2005; Claeys and Vermeire, 2019). Hypoxic injury is considered to play a role in graft rejection through the induction of apoptosis and disruption of graft β-cell functions. Interestingly, it was recently demonstrated in a study that human mesenchymal stem cell (MSC)-originated exosomes can improve tissue resistance to hypoxic environments and therefore protect neonatal porcine islet cells to a notable extent (Nie et al., 2018). While the use of MCS-based platforms has brought significant advances to the field of immunotherapeutics for cancer and autoimmune disease treatments, MSC-derived exosomes are considered to possess superior activities and anti-inflammatory properties and hence are recommended for alternative therapeutic platforms (Askenase, 2020). MSC-derived exosomes can effectively reduce the release of pro-inflammatory cytokines by peripheral blood mononuclear cells (PBMCs), increase the release of IL-10 and TGF-β1 from PBMCs, and consequently promote the immunosuppression potential of Tregs (Meng et al., 2020). Exosomes derived from MSCs can also improve innate immunity by decreasing neuroinflammation and increasing the abundance of Tregs in autoimmune encephalomyelitis mouse models (Riazifar et al., 2019). Owing to their anti-inflammatory and immunomodulatory activities, exosome-based therapy in combination with minimum immunosuppressant consumption is considered to achieve long-term organ survival and prevent future rejection reactions in graft recipients (Monguió-Tortajada et al., 2014). An *in vivo* study investigating the efficacy of human hepatic stem-like cells (HLSCs)-derived EVs in hypoxic rat liver perfusion models observed decreased hepatocyte lysis markers and hepatocyte damage upon perfusing the graft with the aforementioned EVs (Rizza and Catalano, 2018). Similar results were also obtained in regards to lung (Weiss et al., 2017) and heart (Zhao et al., 2019) in previous reports. Another remarkable attribute of exosomes in organ transplantation is related to their ability to monitor the host’s tolerance to kidney (Schröppel and Legendre, 2014), heart (Kennel et al., 2018), liver (Ono et al., 2018), islet (Vallabhajosyula et al., 2017), and lung (Gunasekaran et al., 2018)transplantation.

Besides their promising potential in improving organ survival and transplant success rates, evidence has revealed that exosomes can act as nanoscale biological warehouses that transfer a wide range of biomolecules from their originated cells into other parts. Depending on existing disease states, exosomes can carry disorder-specific biomarkers that can allow them to be used as early-stage prognosis platforms (Hossain et al., 2022; Hsu et al., 2022; Hu et al., 2022; Yi et al., 2022). However, disease-associated biomarkers and proteins carried by exosomes can also partially impair normal physiological signaling pathways and speed up metastasis and tumor angiogenesis by taking part in intercellular crosstalk between tumor cells and nearby stromal cells (Wu et al., 2019a; Wu et al., 2019b; Liu et al., 2022). Accordingly, growth factor-containing exosomes derived from tumor cells are capable of enhancing cancer cell proliferation and differentiation by transferring their parent cells’ information (such as inflammatory markers, growth factors, and miRNAs) to distant malignant cells and hence contribute to the progression of cancer (Maia et al., 2018; Eble and Niland, 2019; Liu et al., 2021).

Depending on pathological conditions, exosomal contents, and originated cells, exosomes may contribute to disease progression or prevent further worsening of existing diseases (Maia et al., 2018; Liu et al., 2021). Accordingly, tumor-released exosomes obtained from leukemia patients have been shown to weaken the host immune system by hindering the activity of natural killer (NK) cells, thus leading to the progression of cancer (Whiteside, 2013). Exosomal glycoprotein-130 (gp130) obtained from breast cancer cells was also observed to contribute to the progression of cancer through the activation of the IL-6/STAT3 pathway in macrophages thus leading to the alteration of normal macrophage phenotypes into pro-cancer phenotypes (Ham et al., 2018). Interestingly, exosomal miR-9 and miR-181a were reported to promote breast cancer through the activation of the JAK/STAT signaling pathway and increasing the production of early myeloid-derived suppressor cells that express significant levels of IL-6 (Jiang et al., 2020). On another note, a group of researchers recently reported that exosomes from IFN-γ-stimulated-thyrocyte cells express inflammatory antigens such as TPO, HSP60, and MHC-II and also activate proinflammatory dendritic cells (DCs) that can trigger the occurrence or development of autoimmune thyroid diseases through complex signaling pathways (Cui et al., 2020).

Elucidating the role of exosomes in attenuating or increasing immune responses during different pathological conditions can serve as an opportunity to shift their impact toward desired outcomes. For instance, exosome engineering, loading with specific biomolecules, and exosome-based drug delivery systems serve as potential immunotherapeutic platforms that are capable of manipulating immune responses in favor of disease treatment (Wang et al., 2015). Owing to their low immunogenicity, nanoscale dimensions, and carrier characteristics, exosomes are now increasingly employed in the development of cancer immunotherapeutics, cancer vaccines, and drug delivery (Yuan et al., 2019; Li et al., 2020b; Lindenbergh et al., 2020; Li et al., 2021; Liang et al., 2021; Liu et al., 2022). Seo Et al., indicated that the administration of exosomes originating from CD8^+^ T cells has significantly reduced metastasis and fibroblastic stroma-mediated tumor invasion in melanoma mouse models (Seo et al., 2018). An interesting report has also revealed that exosomes released by high-affinity cytotoxic T cells (CTLs) in the presence of IL-12 can successfully activate low-affinity CTLs which are of critical importance for cancer immunotherapy (Wu et al., 2019a). Another study investigating the role of exosomes in breast cancer treatment indicated that the utilization of mesothelin (MSLN)-targeted CAR-T cell-originated exosomes successfully inhibited the growth of MSLN-positive cancer cells in triple-negative breast cancer (Yang et al., 2021). An exosome-based immunotherapeutic platform was recently designed to target T-cell CD3 and breast cancer-associated HER2 receptors. Accordingly, promising results were obtained as this cell-derived exosome-based platform displayed significant anti-tumor activities through increasing immunity against HER2-expressing tumor cells both *in vitro* and *in vivo* (Shi et al., 2020).

Serving as one of the most critical first-line defenses of the human body against carcinogenesis and as well as many other disorders, natural killer (NK) cells play a major role in anti-cancer immunity through rapid detection and efficient elimination of oncogenic cells that lack MHC class I antigen expression (Paul and Lal, 2017; Abel et al., 2018; Wu et al., 2019c; Minetto et al., 2019). Similar to almost all other cell types, NK cells have been proven to secrete exosomes that inherit their parent cells’ main markers such as CD56 antibody and FasL killer protein (Fais, 2013; Hosseini et al., 2022). Strikingly, NK cell-derived exosomes have been indicated to elicit anti-tumor and cytolytic activities directly at the tumor site via diffusing into those tissues. Characterization of NK-derived exosomes has demonstrated that LFA-1 and DNAM1, two NK-associated molecules that are crucial for NK cells’ tumor-killing properties, are clearly expressed in them, however, only DNAM1 was shown to play a substantial role in the cytotoxic activities of NK-originated exosomes (Di Pace et al., 2020). As suggested in recent studies, NK-derived exosomes are also capable of discriminating between normal and cancerous cells which allows them to specifically exert their cytotoxic activities on tumor cells without damaging non-tumor cells (Di Pace et al., 2020; Wu et al., 2021; Hosseini et al., 2022). Another study has demonstrated that activated NK-derived exosomes possess miR-186 and exert cytotoxic activities against neuroblastoma cancer cells and also disrupt its TGF-β-dependent immune evasion (Neviani et al., 2019). Lee et al. (Lee et al., 2021), reported that exosomes derived from natural killer cells had successfully reduced tumor size and also attenuated tumorigenesis through decreasing the expression of a potent cancer stem cell marker called CD133 in canine breast cancer models. These findings demonstrate the promising potential of exosomes in anti-cancer immunotherapy. As bio-vehicles that enhance anti-cancer immune responses and transfer inherited biomarkers from their parent cells, it is important to investigate their roles in disease progression in parallel with novel exosome-based therapeutic strategies in order to reach a comprehensive insight into their essence and avoid any unwanted risks that may possibly come with exosome-based immunotherapy platforms (Cheng et al., 2022; Park, 2022).

## 9 Small EVs and the metabolic syndrome: Friend or foe?

Metabolic syndrome is a common metabolic disorder that has become one of the major public health challenges worldwide (Roma-Rodrigues et al., 2014). This syndrome is usually associated with abdominal obesity, hyperglycemia, high blood triglyceride, low high-density lipoprotein (HDL) cholesterol levels, high blood pressure, inflammation, insulin resistance, and increased risk for developing cardiovascular diseases and type 2 diabetes (Yu et al., 2006; Livshts et al., 2015). The parent cell-derived contents of small EVs can help the early identification and diagnosis of metabolic syndrome and its associated conditions, henceforth facilitating monitoring and controlling this disorder. However, recent evidence has indicated that small EVs play a crucial role in the generation and progress of this syndrome (Yu et al., 2006; Li et al., 2017), thus giving rise to questions regarding whether small EVs actually contribute to the progress of metabolic disorders, or they are just products of such pathological conditions. The abundance of EVs is closely related to an individual’s blood pressure, body mass index (BMI), HOMA-B, and HOMA-IR (measurements for the assessment of β-cell function and insulin resistance, respectively) (Yoshinao Kobayashi et al., 1967). Accordingly, the number of plasma EVs is regulated and reduced through losing weight through physical exercise, maintaining a low-calorie diet, and gastrectomy operations (Pardo et al., 2018). Previously, dysregulations in small EV profiles have also been reported in the adipose tissue, liver, and skeletal muscles of patients with metabolic diseases, especially diabetes mellitus and obesity (Sidhom et al., 2020). Furthermore, alterations and dysregulations in small EVs contents (e.g. peptides, nucleic acids, metabolites,…) can affect disease progression or in some cases play a protective role in diabetes and obesity patients. For example, exerkine factors (factors that are released in response to exercise, including peptides, nucleic acids, and metabolites) released from all tissues and organs are capable of mediating signal transduction (Théry et al., 2018) and are reported to have protective effects in diabetic and obese patients (Gardiner et al., 2016; Théry et al., 2018). In the following section, the role of small EVs in several metabolic syndrome conditions is discussed.

### 9.1 Obesity, insulin resistance, and Type 2 diabetes mellitus

Type 2 diabetes mellitus (T2DM) accounts for the majority of diabetes cases (90%–95%) (Livshts et al., 2015) and affects over 350 million people worldwide (Linares et al., 2015; Szatanek et al., 2015). This type of diabetes develops with defected insulin secretion and insulin resistance in patients (Yu et al., 2018), and obese and overweight people or patients with abdominal obesity have a higher risk of suffering from this disorder (Livshts et al., 2015). Moreover, in a wide range of systemic and omics studies diabetic patients have an increased risk of fatality due to cardiovascular diseases in comparison with non-diabetic patients of the same age (Linares et al., 2015; Szatanek et al., 2015), while also suffering life-threatening complications such as diabetic nephropathy (Hashemi et al., 2021).

Adipose tissue has been known to serve as a major regulator of energy levels in living beings by affecting the most important metabolic organs such as the liver, pancreas, kidney, and skeletal muscles (Livshts et al., 2015; Merchant et al., 2017). Therefore, any deficiencies in this tissue’s function can result in disorders in metabolic homeostasis and insulin resistance, which are prevalent in overweight subjects (Li et al., 2017). This tissue can regulate metabolism via a diverse range of pathways throughout which, miRNAs are one of the most crucial components involved. In this state, miRNAs act as hormones even though they have short half-lives and are degraded quickly due to the activity of RNases in natural biofluids (Merchant et al., 2017). Furthermore, the discovery of certain proteins and biomarkers in small EVs reveals their crucial role as mediators of cell communication and disease progress, and also confirms that the evaluation of exosomes in biofluids may be a potential biomarker for screening different diseases.

Evidence shows that obesity is closely linked with increased inflammation as a result of immune system cell recruitment by adipose cells. Many factors such as adipose tissue hypertrophy, lipotoxicity, increased chemokine, and cytokine release by adipocytes promote the recruitment of immune cells (neutrophiles, macrophages, leukocytes) and consequently lead to the escalation of inflammatory responses in the adipose tissue (Bruun et al., 2005; Cinti et al., 2005; Reilly and Saltiel, 2017). One of the important characteristics of adipose tissue is its remarkable endocrine capacity and secreting of various hormones and cytokines (such as leptin, resistin, TNF-alpha, and IL-6) that can escalate inflammation (Fink et al., 2014). On the other hand, macrophages as mononuclear phagocytic immune cells that are abundantly found in adipose tissues (Weisberg et al., 2003), are involved in promoting inflammation by triggering adipocytes to release pro-inflammatory molecules (TNF-alpha, IL-6). Strikingly, myocytes also release excessive amounts of inflammatory cytokines (IL-6, IL-8, IL-15) and myokines (Fink et al., 2014) in obesity thus leading to additional inflammation, damage to skeletal muscle cells, and disrupting endocrine activity (Balaj et al., 2011). This phenomenon is believed to be the result of adipose-skeletal muscle cross-talk in chronic obesity that induces skeletal muscle-site inflammation as a result of elevated chemokine and adipokine secretion into the blood circulation (Collins et al., 2018; Dini et al., 2020).

There is no doubt that obesity, specially viscellar obesity, is closely related to the development of IR and T2DM (Rask-Madsen and Kahn, 2012; Mastrototaro and Roden, 2021). Interestingly, obesity has been associated with increased generation of EVs secreted from endothelial cells (ECs), adipocytes, leukocytes, and platelets (Helal et al., 2011; Stepanian et al., 2013). A previous report has indicated that injection of EVs from metabolic syndrome individuals to healthy mice alters and disrupts aorta responses to acetylcholine (Agouni et al., 2011; Safiedeen et al., 2017), activates Fas/Fas ligand signaling pathways, and therefore triggers ROS-dependent activation of neutral sphingomyelinase (Safiedeen et al., 2017). Obese mice adipose tissue-derived small EVs have been reported to promote macrophage activation and pro-inflammatory cytokine secretion (Deng et al., 2009). Adipose-derived small EVs can also carry microRNAs that negatively affect normal insulin signaling pathways via impairing related mRNA transcripts and increasing pro-inflammatory mediators, thus contributing to the development of T2DM (Thomou et al., 2017).

Numerous studies have aimed to understand the proteins or miRNAs of serum or urine small EVs as predictive and reflective markers of disease development, such as cardiometabolic risk factors (Lobb et al., 2015; Konoshenko et al., 2018) and renal deficiency in diabetic people (Vergauwen et al., 2017; Zhang et al., 2020). miRNA-143 is a nucleic acid marker found in small EVs that targets the liver and induces IR and angiogenesis (Balkom et al., 2013). A recent work has also displayed that when exosomes are harvested from an obese person’s adipose tissue and incubated to A459 cell culture media, they have the ability to alter the expression of main proteins in Wnt/b-catenin and TGF-b signaling pathways, which are involved in inflammation processes (Böing et al., 2014). In a subsequent study, it was demonstrated that the small EVs from obese adipose tissues managed to impair insulin signaling when applied to the hepatocyte cell line HepG2 (Gámez-Valero et al., 2016). Deng *et al* showed that adipose tissue exosomes which were taken from obese mice explants had higher levels of TNF-a, IL-6, glucose intolerance, and insulin resistance compared to non-obese mice and interestingly, these small EVs were observed to cause inflammatory reactions and subsequent obesity-associated insulin resistance in slim mice (Koliha et al., 2016). A study by Hubal and coworkers demonstrated different exosome secretion levels and cargo composition in obese and lean individuals. As stated, the expression of 55 exosome miRNAs were altered in obese cases (Madison et al., 2014).

Successful detection of miRNAs in T2DM clinical samples containing miR-126, miR-26a, miR-326, miR-133b, miR-342, miR-30a, miR-320c, let-7a, and let-7f, … have been reported (Ghosh et al., 2014; Balaj et al., 2015; Zhou et al., 2016; Boriachek et al., 2019), that might also be involved in provoking subsequent pathological conditions (Ghosh et al., 2014; Balaj et al., 2015; Reiner et al., 2017) and an increased risk for the eventual development of coronary artery diseases. It was also stated that one-third of diabetic patients are infected with Diabetic Nephropathy (DN) (a micro angiopathic complication) (Brand et al., 2014). Many studies have shown that in these patients, water channel aquaporins (AQPs) are deregulated (they are in the plasma membranes of epithelial tubular cells) in DN and have been detected in urine exosomes isolated from patients with T2DM (Brand et al., 2014). Moreover, in other studies about diabetic nephropathy, increased amounts of CD41 in exosomes have been observed regarding a patient with T2DM disease, and the association of high CD41 levels with diabetic nephropathy was confirmed (Wang et al., 2013). In another study, Kranendonk *et al.* compared CD14 (this factor is recognized as a member of the immune system) levels in serum exosomes of obese patients with T2DM, with that of normal and healthy people. The results obtained from that study indicated that CD14 levels in the serum of obese patients had significantly decreased in comparison with people without T2DM (Chen et al., 2010).

It was explained that circulating exosomes of patients with T2DM contained lower serpin G1 levels (also known as C1 inhibitor) compared to healthy controls (Kanwar et al., 2014) which is probably because T2DM also happens due to low levels of serpin G1 content in small EVs of obese individuals as it can inhibit several other factors (such as kallikrein and factor IIa) that create disrupted immune responses in T2DM patients (Davies et al., 2012). Moreover, CD41, dipeptidyl peptidase IV (DPPIV), Wilm’s Tumour-1 (WT-1), and alpha-1-microglobulin/bikunin precursor (AMBP) are putative small EV urinary biomarkers that are associated with diabetic nephropathy (Admyre et al., 2003; Eckel et al., 2005; Wu et al., 2017).

### 9.2 Atherosclerosis

Often accompanied by or resulting from metabolic syndrome, atherosclerosis is a potentially life-threatening pathophysiological condition that is characterized by chronic inflammation in coronary arteries due to the accumulation of LDL and apoptic cells that disrupt normal blood flow at that site. The damaged area often undergoes severe inflammation and immune cell recruitment as a result of accumulated LDL oxidation and apoptic cell necrosis (Ley et al., 2011; Bäck et al., 2019; Wolf and Ley, 2019). LDL oxidative takes place as a result of increased reactive oxygen species (ROS) such as superoxide radicals, hydrogen peroxide, and hydroxy radicals that participate in the genesis or progress of various diseases such as cancer (Morry et al., 2017; Solleiro-Villavicencio and Rivas-Arancibia, 2018), gastrointestinal disorders (Mousavi et al., 2020), neurodegenerative diseases (Solleiro-Villavicencio and Rivas-Arancibia, 2018), cardiovascular diseases (Senoner and Dichtl, 2019), and metabolic diseases (Roberts and Sindhu, 2009). When ROS-scavenging enzymes are depleted or do not have enough capacity to reduce ROS, oxidative stress is initiated, and it can trigger inflammatory responses in a specific site. On the other side, endothelial cells contribute to the cleansing of newly-formed plaques in atherosclerosis by autophagy, however, smooth muscles of plaque-containing arteries have been observed to secrete EVs with certain miRNAs that deteriorate autophagic pathways in that area (Li et al., 2016). Interestingly, many EVs secreted by a wide range of immune cells (macrophages, foamy cells, and dendritic cells) might initiate pro-inflammatory responses in endothelial cells through TNF-α mediated mechanisms (Gao et al., 2016; Osada-Oka et al., 2017).

### 9.3 Hepatotoxicity

EVs are considered to play major roles in the development of hepatic diseases (Martínez and Andriantsitohaina, 2017; Shen et al., 2017). Palmitate and stearate are examples of lipotoxic fatty acids that increase the release of small EVs (Hirsova et al., 2016; Kakazu et al., 2016) that enhance inflammation and TNF-related apoptosis in the liver due to their high ceramide levels. The production of EVs from hepatocytes, adipocytes, and myocytes has been shown to increase under the influence of saturated fatty acids as a result of hyperlipidemia. EVs released upon hyperlipidemic conditions mainly carry lipids and ceramides to macrophages and myocytes (Aswad et al., 2014; Koeck et al., 2014; Hirsova et al., 2016) and induce inflammation in hepatic cells (Koeck et al., 2014), which can eventually lead to the occurrence of non-alcoholic steatohepatitis and non-alcoholic fatty liver disease (NAFLD). miRNA-122 and miRNA-33a/b are biomarkers found in small EVs that target the liver and liver macrophages in respective order, increase the risk of hepatocellular carcinoma and disrupt normal cholesterol metabolism (Balkom et al., 2013). miRNA-103/107 are carried by small EVs and increase adipogenesis in hepatic cells (Taylor and Gercel-Taylor, 2008). Interestingly, during hepatic fibrosis exosomes transport miRNA-214 from hepatic stellate cells toward hepatocytes in order to inhibit certain connective growth factors such as CCN2 and transfer fibrotic signals between cells (Lobb et al., 2015). On the other hand, ischemic/reperfusion injuries can partially increase hepatic regeneration through increasing certain exosomes which possess hepatic proliferative properties (Konoshenko et al., 2018). Last but not least, miRNA-199 is a liver-targeting biomarker found in small EVs and acts as a common biomarker for the identification of NAFLD (Povero et al., 2014). All in all, it can be concluded that exosomes can be utilized to identify hepatic damage, ameliorate hepatic injury conditions, and also create inflammatory environments that contribute to the deterioration of liver normal physiological conditions.

## 10 Small EVs mediation in gut microbiota-host cross-communication

The gut microbiota is a crucial factor for obtaining metabolic health (Dehghanbanadaki et al., 2021). Reduction of intestinal microbiota has also been linked with increased risks for hepatotoxicity, obesity, and metabolic syndrome (Ghosh et al., 2014). Small EVs, as mediators of cell-to-cell crosstalk, represent a platform for host-microbiota communication that may possess the potential for altering the normal gut microbiome composition and lead to the development of metabolic syndrome (Ghosh et al., 2014). For example, EVs produced and secreted by the gut microbiota are capable of disrupting normal glucose metabolism, impairing insulin sensitivity *in vitro* and *in vivo*, (Zhang et al., 2015b; Choi et al., 2015; Badi et al., 2017), increasing inflammatory cell deployment, and altering normal metabolic functions (Khalyfa et al., 2017). A study by Liu et al., 2016 has revealed that declined levels of exosomal miRNA are associated with deteriorated colitis and altered microbiome function. The small EVs that mediate the bidirectional crosstalk between the gut microbiota and host can provide valuable information regarding the individual’s metabolic state through the assessment of exosomal contents detected in feces.

## 11 Future directions

Providing the opportunity to intentionally target intracellular crosstalk and modulate different diseases at the cell-scale level, small EVs have recently gained notable attention globally. Exosome manipulation and engineering, transfer of small EVs from healthy individuals to unhealthy patients (such as in obesity and hyperlipidemia), and even small EV-based drug delivery platforms are some of the most outstanding properties to which exosomes owe their ever-growing clinical value. As briefly discussed in the aforementioned sections, it is believed that small EVs may be potentially capable of accelerating disease progression as they are key contributors to cell-to-cell or organ-to-organ crosstalk. It is also expected that EVs that are involved in disease pathogenesis may also have synergistic effects when accompanied by other risk factors or impaired signaling pathways, however, the lack of data has made it rather difficult to draw certain conclusions regarding the precise roles of small EVs in the initiation or progression of metabolic diseases, either by themselves or in combination with other contributing factors. Nevertheless, it has now become a general consensus that the biological roles of exosomes reach beyond our previous knowledge of them as mere diagnostic biomarkers. It is crucial to keep in mind that the double-edged nature of small EVs in the progress/initiation and treatment of diseases requires unbiased perspectives during the development of new exosome-based therapeutic strategies and techniques. It is also notable to mention that the consumption of different drugs may potentially alter small EVs production, secretion, and composition and thus be exploited as a determinative factor to assess the effects of therapeutic agents upon administration, however, this hypothesis requires extensive investigations regarding various medicinal compounds and molecules.

## Conclusion

In this review, small EVs’ biogenesis, characterization, isolation methods, and potential applications as diagnostic biomarkers and drug/gene delivery platforms were reviewed. Regarding the isolation of small EVs, different approaches were explained and briefly compared depending on their cost, speed, human involvement, required time, exosome recovery, purity, yield, and efficiency. Owing to their exquisite properties and intercellular crosstalk mediation, it is now believed that small EVs can be manipulated to target desired cellular signaling pathways and play therapeutic roles in the improvement of pathological conditions. Moreover, their inherent vehicle-like characteristics have introduced exosomes as advantageous drug/gene delivery platforms with low immunogenicity. However, current literature also suggests that small EVs are capable of contributing to disease progression or initiation, and numerous studies have revealed their participation in the initiation or deterioration of metabolic disorders. Falling under the cluster of metabolic syndrome, several conditions such as obesity, type 2 diabetes mellitus (T2DM), atherosclerosis, and several hepatic injuries are some of the most life-threatening pathological disorders through which exosomes play important mediatory roles. As previously discussed in comprehensive detail, the extent to which exosomes contribute to the pathogenesis of the metabolic syndrome is unknown, as the interrelated pathways and potential synergism between different risk factors and small EVs are complicated factors that require further investigations to be fully explored.
